# Are Athletes Healthy?

**Published:** 1899-04

**Authors:** 


### Are Athletes Healthy?

This question is propounded by *The Hospital*, which at once proceeds to answer it in a smewhat equivocal manner. It depends altogether, we are told, on what you call an athlete. In general, athletes are healthy, but not because they are athletes—the sequence of cause and effect is the other way about. Says the journal just mentioned:

"If by an 'athlete' is meant a 'trained' man, one answer to the question is immediately obvious, viz, that whether he is healthy or not, his health is not due to his training. If a demand arose, as in fact it does arise in military service, for men of endurance, of considerable muscular strength, of great and varied digestive capacity, able to bear exposure, starvation, and attacks of infectious illness with the least possible disturbance, and all the time fit for the exercise of the highest intelligence, then a training which produced such men would no doubt be conducive to health. But the training which athletes undergo does not even aim at such a condition. Its whole object is to produce the greatest possible output of muscular energy, to apply it with the utmost exactitude in a given direction, and at the same time to develop a state of body as to weight, etc., which shall conform to certain rules. The routine by which these results can be most readily obtained is, no doubt, effectual for its purpose—but that purpose is not the production of health.

"An athlete, although often a very healthy man, by no means obtains his health by virtue of his training. Quite the opposite, in fact. Those who by build and constitution require severe regimen and training to make them fit for athletic work, and who drop out of condition as soon as they cease to train, really suffer in health from the process—in the case of jockeys and others who train for weight, they often do suffer very severely —while others who are always fairly fit may even benefit by the course. But when we come to consider what, in these record-breaking days, an athlete has to do in his 'spurts' and in his practice, we are quite clear that the life of an athlete does not tend to health. We quite admit that a large number of healthy middle-aged, and even old men, are to be met with who have ardently indulged in athletics in their earlier years, but the fact that athletic sports are chiefly attractive to the strong and healthy makes the athletes so select a class that no comparison can be fairly drawn between them and the average man. Athletes are healthy because they are select, not because they are athletic. On the other hand, when we come to compare them with the average men we must remember that average men have many vices. We would not urge a young man to become an athlete in the modern sporting sense, but perhaps he had better even do that, than oscillate between the office, the billiard-room and the whiskey bar."

There is something radically and wickedly wrong about the training of college athletes when its good effects not only culminate on a single day, but are followed by a rapid deterioration of strength when that day passes. Such methods are all right when applied to race horses and chrysanthemums, whose fate after they win the covoted stake or prize for their owners is of no particular consequence, but in the case of young men who have other work to do in the world, the attainment of prowess so evanescent and expensive is obviously a mistake.—*Exchange.*

